# Supplementary material for: Type-I hyperbolic metasurfaces for highly-squeezed designer polaritons with negative group velocity
Source: Nat Commun. 2019 May 1;10:2002. doi: 10.1038/s41467-019-10027-0 (PMC6494850; doi:10.1038/s41467-019-10027-0)
Supplement: Supplementary file 1 — Supplementary Information [file 41467_2019_10027_MOESM1_ESM.pdf]

Supplementary Information

Type-I hyperbolic metasurfaces for highly-squeezed designer polaritons with negative group velocity

Yang *et al*.

Supplementary Note 1: From the type-I hyperbolic metasurface to the meta-ribbon.

We have done simulations to show the relation between the type-I hyperbolic metasurface and the meta-ribbon (Supplementary Figure 1). We gradually increase *b* in Supplementary Figure 1, and consider the dispersion of the designer polaritons propagating in the y-direction. The meta-ribbon can be treated as an extreme case when *b* goes to infinity. In Supplementary Figure 1, we show the dispersions of type-I hyperbolic metasurfaces with different *b* and find that when *b* is larger than 40.2 mm, the dispersion of the type-I hyperbolic metasurface is very close to that of the meta-ribbon. One can see that the meta-ribbon inherits the properties of the type-I hyperbolic metasurface, i.e., extremely high effective refractive index, and ultra-large negative group velocity of the designer polaritons. Therefore, the meta-ribbon can be considered as a special case of the type-I hyperbolic metasurface.

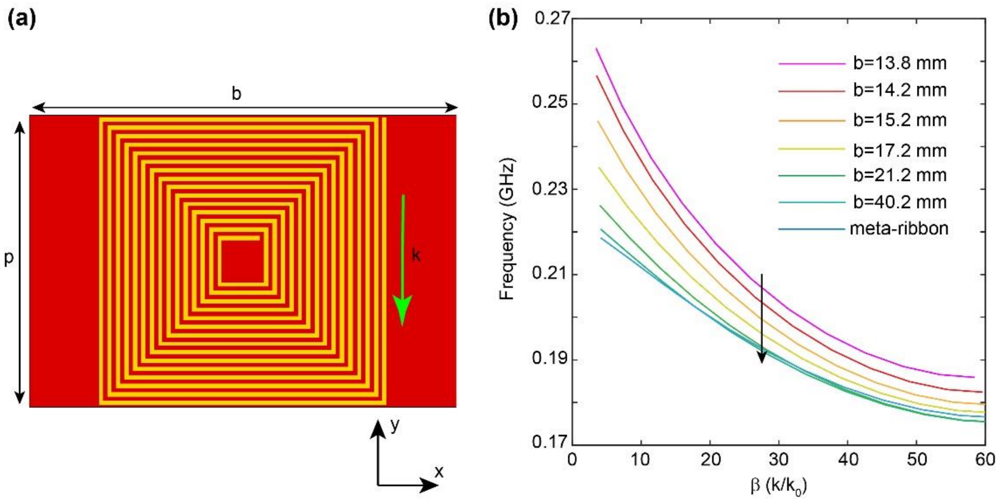

Supplementary Figure S1. From the type-I hyperbolic metasurface to the meta-ribbon. (a) A unit cell of the type-I hyperbolic metasurface. (b) Dispersions of the type-I hyperbolic metasurfaces with different *b* and the meta-ribbon, respectively. Here, we only change *b* and keep the other parameters the same.

Supplementary Note 2: Design of a far-infrared type-I hyperbolic metasurface.

Here, we show the possibility of realizing a type-I hyperbolic metasurface at far-infrared frequencies. The designed far-infrared type-I hyperbolic metasurface is as shown in

Supplementary Figure 2(a), where the yellow region is silver. In the simulation, the silver is described by the well-known Drude model

$$\varepsilon(\omega) = 1 - \frac{\omega_p^2}{\omega(\omega + i\nu)}, \tag{1}$$

with plasma frequency ω_p = 1.37×10^{16} s^{-1} and collision frequency ν= 3×10^{13} s^{-1}. The dispersion of the designer polaritons on the designed metasurface is shown in Supplementary Figure 2(c). The operational frequency is from 2.65 THz to 3.4 THz, which is in the regime of far-infrared frequency (0.3 THz to 20 THz). Besides, the figures of merit (Re(k)/Im(k)) range from 19.2 to 22, comparable with that of h-BN-encapsulated graphene[1]; the squeezing factor (k/k_0) reaches up to 20.

When increasing the numbers of turns, the maximal squeezing factor increases while the figure of merit decreases due to the metallic loss (Supplementary Figure 2(d)). Therefore, there is a trade-off between the maximal squeezing factor and the figure of merit. From Supplementary Figure 2(d), one can find that the limitation of the maximal squeezing factor is about 46, which is comparable with that of h-BN-encapsulated graphene[2].

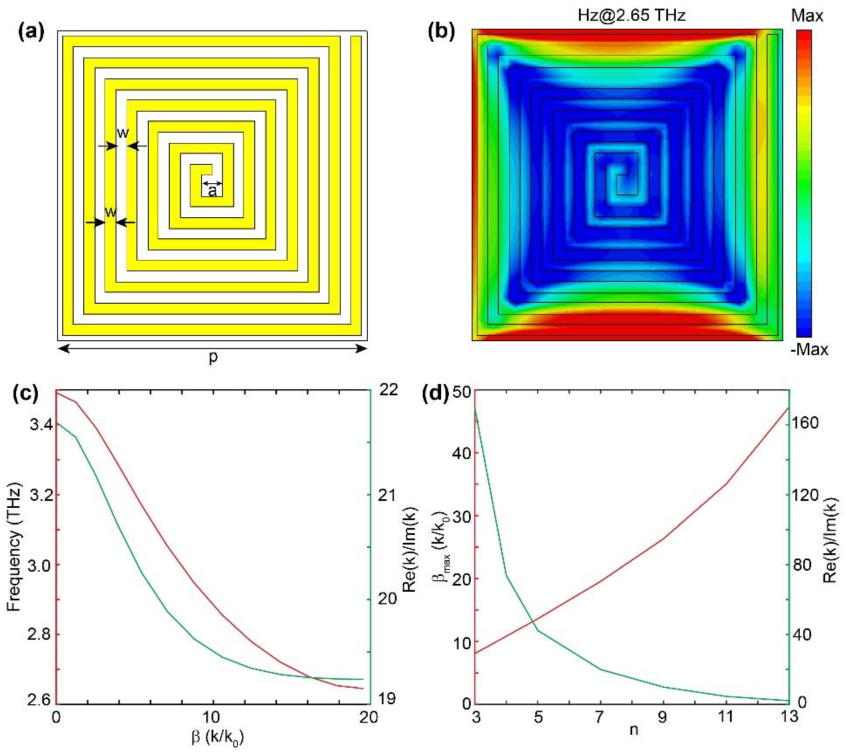

Supplementary Figure S2. Design of a far-infrared type-I hyperbolic metasurface. (a) A unit cell of a far-infrared type-I hyperbolic metasurface. The yellow region is silver. Here, w=0.1 um; a=0.2 um; n=7; p=2.9 um; and the thickness of the silver is 0.1 um. (b) Z-oriented magnetic field distributions at 2.65 THz. (c) Dispersion and figures of merit (Re(k)/Im(k)) of the designed far-infrared type-I hyperbolic metasurface, respectively. (d) Maximal squeezing factors and the corresponding figures of merit of the far-infrared type-I hyperbolic metasurfaces as a function of turn numbers.

Supplementary references

1. Low, T. *et al.* Polaritons in layered two-dimensional materials. *Nature Mater.* **16**, 182-194 (2017).
2. Basov, D. N., Fogler, M. M. & Garcia de Abajo, F. J. Polaritons in van der Waals materials. *Science* **354**, aag1992 (2016).
